# Supplementary material for: Sequential role of RAD51 paralog complexes in replication fork remodeling and restart
Source: Nat Commun. 2020 Jul 15;11:3531. doi: 10.1038/s41467-020-17324-z (PMC7363682; doi:10.1038/s41467-020-17324-z)
Supplement: Supplementary file 1 — Supplementary Information [file 41467_2020_17324_MOESM1_ESM.pdf]

Sequential role of RAD51 paralog complexes in replication fork remodeling and restart

Berti/Teloni et al., *Nature Communications* 2020

Supplementary Information

Supplementary Figures 1-4

Supplementary Tables 1-2

Supplementary Figures

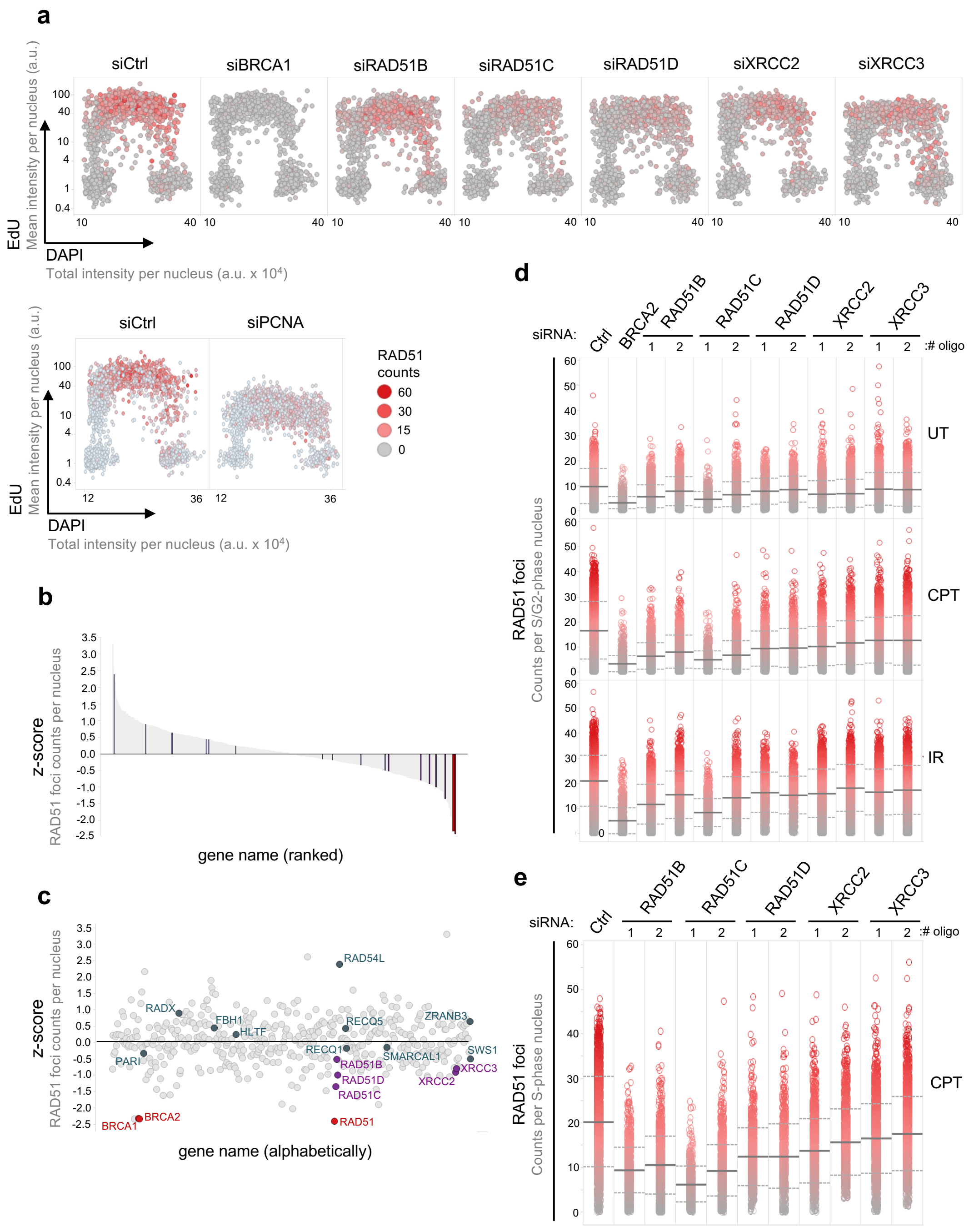

Supplementary Figure 1. QIBC-based screen identifies RAD51 paralogs as important modulators of RAD51 foci formation upon mild replication stress (**a**) Pooled QIBC screen data for siRNAs targeting the indicated genes together with corresponding control conditions (Ctrl). Cell cycle profiles based on DNA content and EdU incorporation and cell cycle distribution of RAD51 foci are shown. **(b)** and **(c)** z-scores for known regulators of replication fork reversal (marked in green), the classical RAD51 paralogs (marked in purple) and positive controls (marked in red), based on data in Supplementary Tables 1 and 2. **(d)** Screen validation in U2OS cells treated with two independent siRNAs against each indicated target gene in otherwise unchallenged conditions (untreated, UT), upon CPT exposure (50 nM, 45 min), or upon IR (5 Gy, 2 hr recovery). RAD51 foci counts per S/G2-phase nucleus, based on EdU incorporation, are shown. (**e**) Effect of RAD51 paralog downregulation on RAD51 foci formation in S-phase U2OS cell, treated with CPT (50 nM for 45 min; red data points in Fig. 1f)**.** Source data are provided in the Source data file.

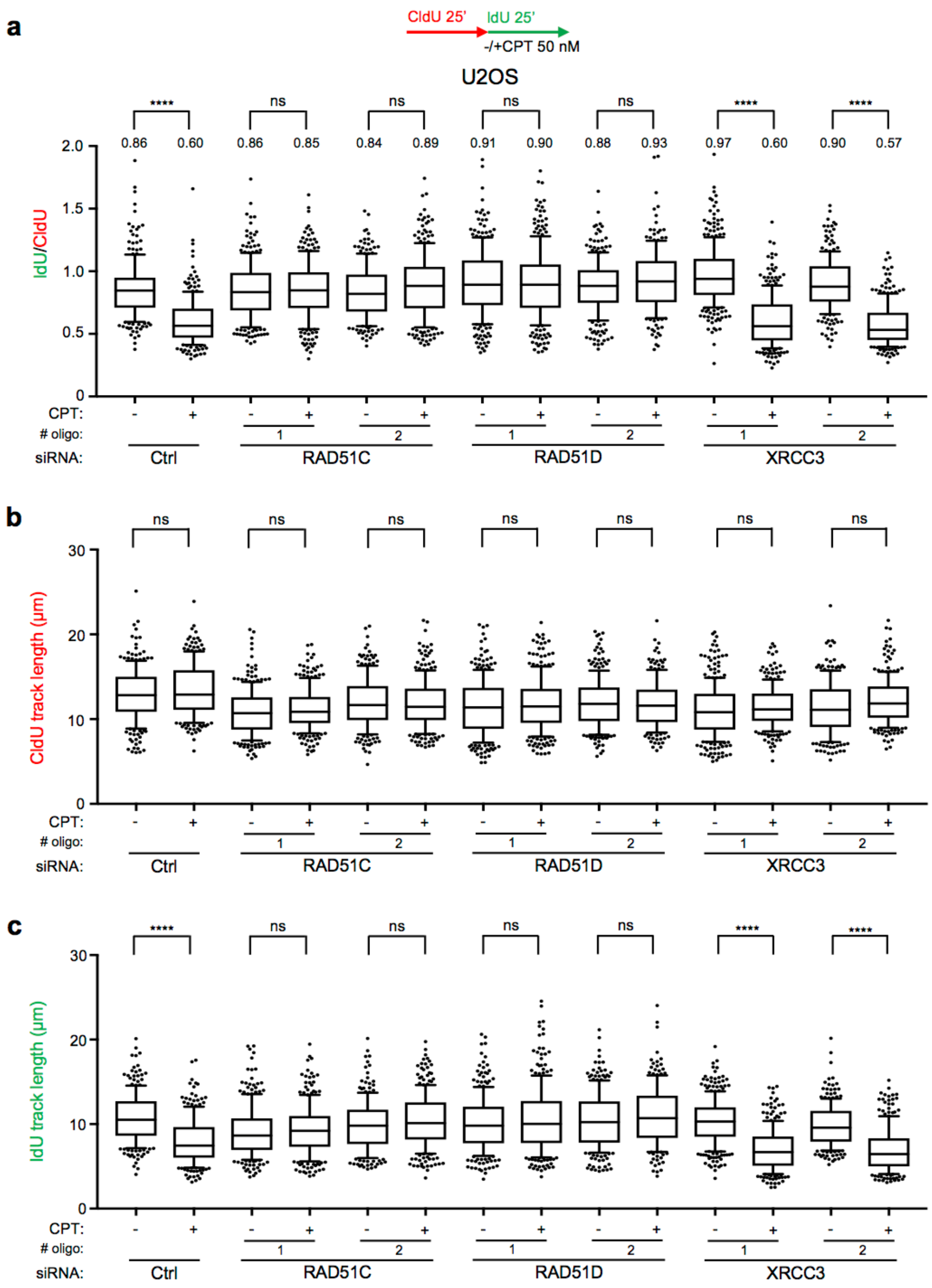

Supplementary Figure 2. See next page for legend

d

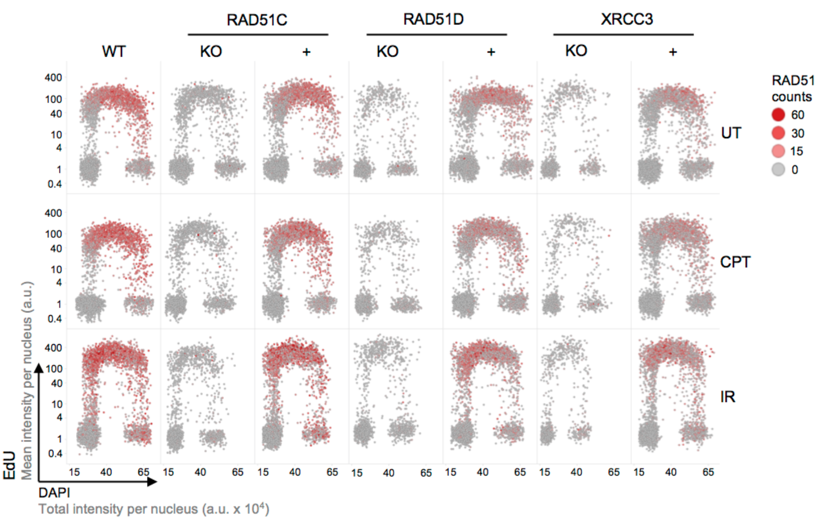

e

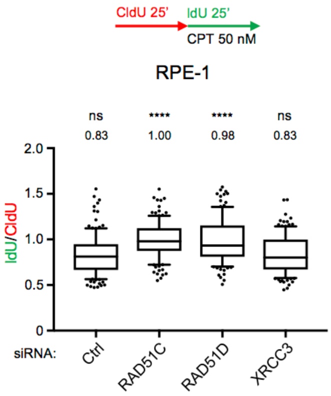

Supplementary Figure 2. BCDX2 paralog complex, but not CX3, promotes replication fork slowing upon mild CPT treatment (a-c) DNA fiber analysis of U2OS cells transfected for 48 hr with a control siRNA (siCtrl) or with siRNAs targeting RAD51C, RAD51D and XRCC3 to investigate replication fork progression. Top: schematic of the CldU/IdU pulse-labeling protocol used to evaluate fork progression upon optional 50 nM CPT treatment. **a**) IdU/CldU track length ratios, the numbers indicate the mean value; **b**) CldU track length; **c**) IdU track length. A minimum of 150 forks was scored. Bounds of box are 25th to 75th percentile, center shows the median, whiskers indicate the 10-90 percentiles, data outside this range is drawn as individual dots. Statistical analysis: Kruskal-Wallis test; ns, not significant; **** p value < 0.0001. Experiment was performed twice yielding similar results. (**d**) U2OS wildtype (WT) or RAD51C-, RAD51D- and XRCC3-KO cells, without or with reconstitution of the respective protein (+), were tested for cell cycle resolved RAD51 foci formation by QIBC. Cells were assessed in unchallenged conditions (untreated, UT), upon CPT exposure (50 nM, 45 min), or upon IR (5 Gy, 2 hr recovery). Cell cycle profiles based on DNA content and EdU incorporation and cell cycle distribution of RAD51 foci are shown. **(e)** DNA fiber analysis of RPE-1 cells transfected for 48 hr with a control siRNA (siCtrl) or with siRNAs targeting RAD51C, RAD51D and XRCC3 to investigate replication fork progression. Top: schematic of the CldU/IdU pulse-labeling protocol used to evaluate fork progression upon 50 nM CPT treatment. Bottom: IdU/CldU ratio is plotted as a read-out of fork progression. The numbers indicate the mean value. A minimum of 150 forks was scored. Bounds of box are 25th to 75th percentile, center shows the median, whiskers indicate the 10-90 percentiles, data outside this range is drawn as individual dots. Statistical analysis: Kruskal-Wallis test; ns, not significant; **** p value < 0.0001. Experiment was performed twice yielding similar results. Source data for a-e are provided in the Source data file.

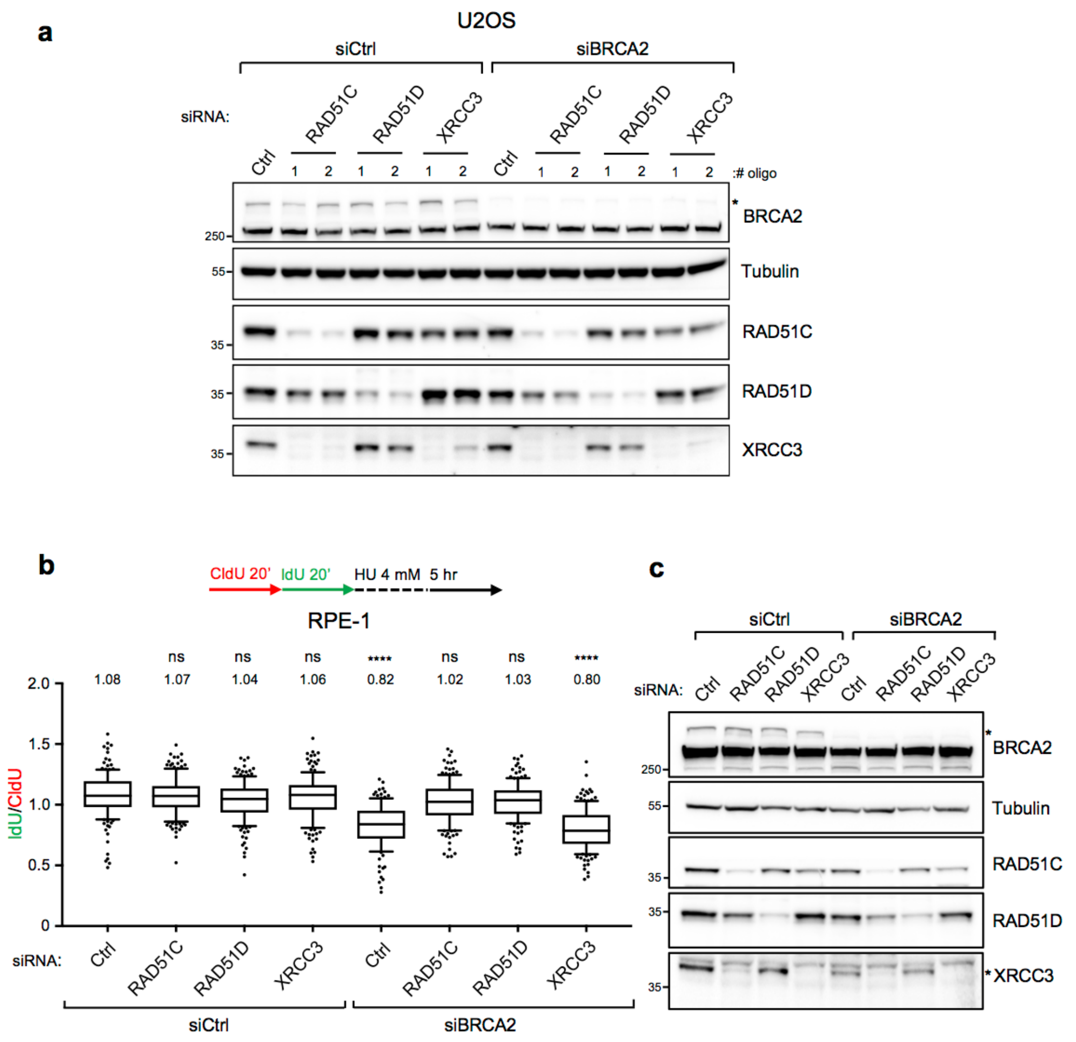

Supplementary Figure 3. BCDX2 complex, but not CX3, promotes fork degradation in BRCA2-depleted cells (**a**) Western blot analysis relative to Fig 3a, b. Ctrl, control siRNA. Tubulin, loading control; *, specific band. In (a) and (c) multiple gels/blots were processed in parallel, ensuring equal and comparable loading across gels. Experiment was performed twice yielding similar results. (**b**) DNA fiber analysis of RPE-1 cells double-transfected with a control siRNA (Ctrl) or with siRNAs targeting RAD51C, RAD51D and XRCC3 for 60 hr and with BRCA2 siRNA for 48 hr. Top: schematic of the CldU/IdU pulse-labeling protocol used to evaluate fork degradation upon HU treatment (4 mM, 5 hr). Bottom: IdU/CldU ratio is plotted as a read-out of fork degradation. The numbers indicate the mean value. A minimum of 150 forks was scored in two independent experiments yielding similar results. Bounds of box are 25th to 75th percentile, center shows the median, whiskers indicate the 10-90 percentiles, data outside this range is drawn as individual dots. Statistical analysis: Kruskal-Wallis test; ns, not significant; **** p value < 0.0001. (**c**) Western blot analysis relative to Fig S3b. Ctrl, control siRNA. Tubulin, loading control; *, specific band. Experiment was performed twice yielding similar results. Source data for a-c are provided in the Source data file.

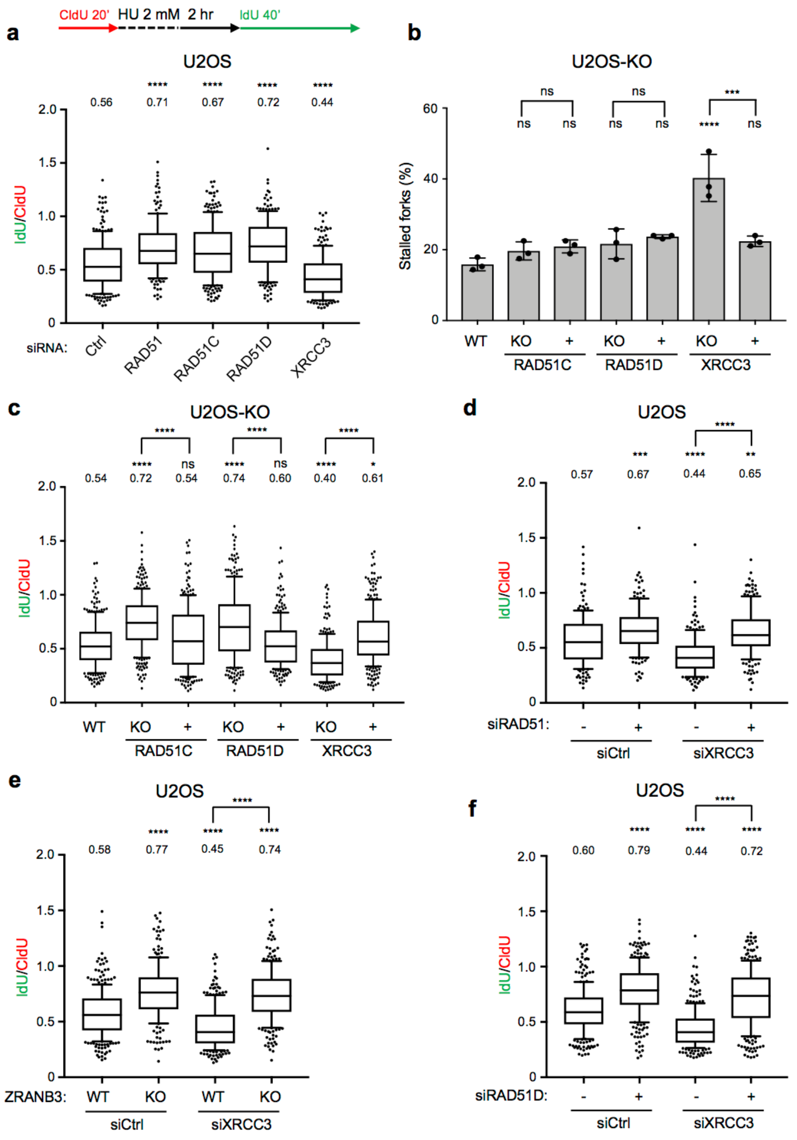

Supplementary Figure 4. CX3 complex promotes reversed fork restart in U2OS cells (**a**) DNA fiber analysis relative to Fig 4a. Top: schematic CldU/IdU pulse-labeling protocol used to evaluate fork restart upon HU treatment (2 mM, 2 hr). Bottom: IdU/CldU ratio is plotted as a read-out of fork restart velocity. Numbers indicate the mean value. A minimum of 150 forks was scored in three independent experiments yielding similar results. Bounds of box are 25th to 75th percentile, center shows the median, whiskers indicate the 10-90 percentiles, data outside this range is drawn as individual dots. Statistical analysis: Kruskal-Wallis test; ns, not significant; **** p value < 0.0001. (**b**) DNA fiber analysis of KO and reconstituted (+) U2OS cells to investigate replication fork restart upon HU treatments, following the labeling protocol in (a). Graph-bar depicts mean and SD of fork stalling frequency from three independent experiments. Statistical analysis: one-way ANOVA followed by Bonferroni test; ns, not significant; *** p value = 0.0003; **** p value < 0.0001. (**c**) DNA fiber analysis relative to Fig S4b. IdU/CldU ratio is plotted as read-out of fork restart velocity. Numbers indicate the mean value. A minimum of 150 forks was scored in three independent experiments yielding similar results. Bounds of box are 25th to 75th percentile, center shows the median, whiskers indicate the 10-90 percentiles, data outside this range is drawn as individual dots. Statistical analysis: Kruskal-Wallis test; ns, not significant; * p value = 0.0465; **** p value < 0.0001. (**d**), (**e**) and (**f**) DNA fiber analysis relative to Fig 4b, 4c and 4d respectively. IdU/CldU ratio is plotted as read-out of fork restart velocity. Numbers indicate the mean value. A minimum of 150 forks was scored in two independent experiments yielding similar results. Bounds of box are 25th to 75th percentile, center shows the median, whiskers indicate the 10-90 percentiles, data outside this range is drawn as individual dots. Statistical analysis: Kruskal-Wallis test; ns, not significant; ** p value = 0.0048; *** p value = 0.0001; **** p value < 0.0001. Source data for a-f are provided in the Source data file.

a

siRNAs:	Ctrl	RAD51C	RAD51D	XRCC3
%RF<br>Exp #1	25.8<br>(81)	5.6<br>(71)	2.9<br>(70)	41<br>(78)
%RF<br>Exp #2	29.6<br>(71)	11.2<br>(80)	9.3<br>(75)	30.5<br>(82)
%RF<br>Exp #3	30.8<br>(78)	3.7<br>(80)	7.9<br>(89)	33.7<br>(81)

b

siRNAs:	Ctrl	Ctrl<br>+ Mirin	BRCA2	BRCA2<br>+ Mirin	RAD51C	RAD51C<br>+ Mirin	XRCC3	XRCC3<br>+ Mirin
%RF<br>Exp #1	23.1<br>(78)	22.7<br>(75)	10.7<br>(79)	21.1<br>(76)	5.7<br>(70)	9.5<br>(85)	24.3<br>(70)	20.8<br>(80)
%RF<br>Exp #2	26.8<br>(71)	19.2<br>(73)	10.7<br>(75)	23.9<br>(77)	5.5<br>(73)	5.1<br>(78)	20.2<br>(84)	21.1<br>(76)
%RF<br>Exp #3	24.7<br>(73)	24.8<br>(71)	3.5<br>(100)	26.7<br>(80)	9.9<br>(76)	5.5<br>(72)	30.5<br>(70)	27.1<br>(70)

Supplementary Table 1. Electron microscopy summary data. (a) Frequency of observed reversed forks (% RF) in three independent experiments relative to Figure 2g. **(b)** Frequency of observed reversed forks (% RF) in three independent experiments relative to Figure 3d. Number of analyzed molecules is indicated in brackets.

siRNA	SOURCE	IDENTIFIER
SilencerSelect negative control No.1 siRNA (siCtrl)	ThermoFisher	Cat# 4390843
SilencerSelect siRNA against RAD51B #1	ThermoFisher	Cat# ID s11740
SilencerSelect siRNA against RAD51B #2	ThermoFisher	Cat# ID s11742
SilencerSelect siRNA against RAD51C #1	ThermoFisher	Cat# ID s11738
SilencerSelect siRNA against RAD51C #2	ThermoFisher	Cat# ID s11737
SilencerSelect siRNA against RAD51D #1	ThermoFisher	Cat# ID s11743
SilencerSelect siRNA against RAD51D #2	ThermoFisher	Cat# ID s11744
SilencerSelect siRNA against XRCC2 #1	ThermoFisher	Cat# ID s14944
SilencerSelect siRNA against XRCC2 #2	ThermoFisher	Cat# ID s14945
SilencerSelect siRNA against XRCC3 #1	ThermoFisher	Cat# ID s14947
SilencerSelect siRNA against XRCC3 #2	ThermoFisher	Cat# ID s14948
SilencerSelect siRNA against BRCA2	ThermoFisher	Cat# ID s2083
Microsynth siRNA against RAD51 (F_1)<br>5'-GACUGCCAGGAUAAAGCUUdTdT-3'	Microsynth	N/A
Microsynth siRNA against Luciferase (siLUC)<br>5'-CGUACGCGGAAUACUUCGAUUdTdT-3'	Microsynth	N/A
Microsynth siRNA against BRCA2 (siBRCA2)<br>5'-UUGACUGAGGCUUGCUCAGUUdTdT-3'	Microsynth	N/A

Supplementary Table 2. Oligonucleotides used in this study.
