# Supplementary material for: Sequential role of RAD51 paralog complexes in replication fork remodeling and restart
Source: Nat Commun. 2020 Jul 15;11:3531. doi: 10.1038/s41467-020-17324-z (PMC7363682; doi:10.1038/s41467-020-17324-z)
Supplement: Supplementary file 3 — Description of Additional Supplementary Files [file 41467_2020_17324_MOESM3_ESM.pdf]

Description of Additional Supplementary Files

File Name: Supplementary Data 1
Description: z-score based on RAD51 foci counts in S-phase cells

File Name: Supplementary Data 2
Description: Average number of RAD51 foci for each siRNA in S-phase cells
