# Supplementary material for: Sequential role of RAD51 paralog complexes in replication fork remodeling and restart
Source: Nat Commun. 2020 Jul 15;11:3531. doi: 10.1038/s41467-020-17324-z (PMC7363682; doi:10.1038/s41467-020-17324-z)
Supplement: Supplementary file 6 — Reporting Summary [file 41467_2020_17324_MOESM6_ESM.pdf]

nature research

Corresponding author(s): MASSIMO LOPES
MATTHIAS ALTMEYER

Last updated by author(s): May 22, 2020

Reporting Summary

Nature Research wishes to improve the reproducibility of the work that we publish. This form provides structure for consistency and transparency in reporting. For further information on Nature Research policies, see our Editorial Policies and the Editorial Policy Checklist.

Statistics

For all statistical analyses, confirm that the following items are present in the figure legend, table legend, main text, or Methods section.

n/a	Confirmed	
☐	☒	The exact sample size (*n*) for each experimental group/condition, given as a discrete number and unit of measurement
☐	☒	A statement on whether measurements were taken from distinct samples or whether the same sample was measured repeatedly
☐	☒	The statistical test(s) used AND whether they are one- or two-sided<br>*Only common tests should be described solely by name; describe more complex techniques in the Methods section.*
☒	☐	A description of all covariates tested
☐	☒	A description of any assumptions or corrections, such as tests of normality and adjustment for multiple comparisons
☐	☒	A full description of the statistical parameters including central tendency (e.g. means) or other basic estimates (e.g. regression coefficient) AND variation (e.g. standard deviation) or associated estimates of uncertainty (e.g. confidence intervals)
☐	☒	For null hypothesis testing, the test statistic (e.g. *F*, *t*, *r*) with confidence intervals, effect sizes, degrees of freedom and *P* value noted<br>*Give P values as exact values whenever suitable.*
☒	☐	For Bayesian analysis, information on the choice of priors and Markov chain Monte Carlo settings
☒	☐	For hierarchical and complex designs, identification of the appropriate level for tests and full reporting of outcomes
☒	☐	Estimates of effect sizes (e.g. Cohen's *d*, Pearson's *r*), indicating how they were calculated

Our web collection on statistics for biologists contains articles on many of the points above.

Software and code

Policy information about availability of computer code

Data collection	QIBC data: Olympus ScanR Image Analysis Software version 3.0.0; Electron microscopy: DigitalMicrograph Version 1.83.842 (Gatan, Inc.); Metaphase aqcuisition: Leica Application Suite X 3.6.0.20104; DNA fiber imaging:
Data analysis	Color-coded scatterplots: Spotfire data visualization software version 7.0.1 (TIBCO); DNA fiber, EM and metahpase image analysis: ImageJ software64; Graph Pad Prim version 7 for statistical analysis of numerical data.

For manuscripts utilizing custom algorithms or software that are central to the research but not yet described in published literature, software must be made available to editors and reviewers. We strongly encourage code deposition in a community repository (e.g. GitHub). See the Nature Research guidelines for submitting code & software for further information.

Data

Policy information about availability of data

All manuscripts must include a data availability statement. This statement should provide the following information, where applicable:

- Accession codes, unique identifiers, or web links for publicly available datasets
- A list of figures that have associated raw data
- A description of any restrictions on data availability

Source data underlying Figures 1a-h; 2b-g; 3a-d; 4a-d; 5a; S1a+d; S2a-e; S3a-c; S4a-f are provided in the source data file. All other original microscopy images will be made available upon reasonable request.

Field-specific reporting

Please select the one below that is the best fit for your research. If you are not sure, read the appropriate sections before making your selection.

☒ Life sciences ☐ Behavioural & social sciences ☐ Ecological, evolutionary & environmental sciences

For a reference copy of the document with all sections, see nature.com/documents/nr-reporting-summary-flat.pdf

Life sciences study design

All studies must disclose on these points even when the disclosure is negative.

Sample size	Sample size for all experiments shown (electron microscopy, n>70 in 3 independent experiments; DNA fibers, n>150 in 2 or more independent experiments; metaphase spreads, n>50 in 3 independent experiments) was chosen to obtain statistical power, in conformity to accepted standard sample size in a number of previous publications using these approaches:<br><br>Mijic et al., Nat Commun., DOI: 10.1038/s41467-017-01164-5<br>Vujanovic et al., Mol Cell, DOI: 10.1016/j.molcel.2017.08.010<br>Mutreja et al., Cell Rep., DOI: 10.1016/j.celrep.2018.08.019
Data exclusions	No data were excluded from the analysis.
Replication	For all experiments, the number of biological replicates is indicated and, without any exception, reproduced the representative data shown in the figures.
Randomization	We were working with asynchronously cycling cell populations or individual DNA replication molecules from these cell populations, hence further randomization was not necessary for our approaches.
Blinding	Individual repetitions for Metaphase Spreading, DNA fiber analysis and Electron Microscopy were blinded to the investigators.<br>For the automated QIBC screen blinding was not necessary due to its intrinsically unbiased nature.

Reporting for specific materials, systems and methods

We require information from authors about some types of materials, experimental systems and methods used in many studies. Here, indicate whether each material, system or method listed is relevant to your study. If you are not sure if a list item applies to your research, read the appropriate section before selecting a response.

Materials & experimental systems

n/a	Involved in the study	
☐	☒	Antibodies
☐	☒	Eukaryotic cell lines
☒	☐	Palaeontology and archaeology
☒	☐	Animals and other organisms
☒	☐	Human research participants
☒	☐	Clinical data
☒	☐	Dual use research of concern

Methods

n/a	Involved in the study	
☒	☐	ChIP-seq
☒	☐	Flow cytometry
☒	☐	MRI-based neuroimaging

Antibodies

Antibodies used	Primary antibodies:<br>RAD51B mouse (sc-377192, Santa Cruz Biotechnology); RAD51C rabbit (ab95069, Abcam); RAD51D rabbit (ab202063, Abcam); XRCC2 mouse (sc-365854, Santa Cruz Biotechnology); XRCC3 mouse (sc-271714, Santa Cruz Biotechnology); BRCA2 mouse (OP-95, EMD Millipore); RAD51 rabbit (Bioacademia 70-002); ZRANB3 rabbit (23111-1-AP, Proteintech); KU70 mouse (ab202022, Abcam); α-Tubulin mouse (T9026, Sigma-Aldrich); Rabbit polyclonal RAD51 antibody (Bioacademia 70-002); CldU (ab6326, Abcam, rat); BrdU/IdU (347580, Becton Dickinson, mouse)<br><br>Secondary antibodies:<br>Cy3 donkey anti-rat (712-166-153, LubioScience); Alexa Fluor 488 goat anti-mouse (A11001, ThermoFisher); ECL anti-rabbit (NA934, Sigma); ECL anti-mouse (NA931, Sigma)
Validation	All antibodies used in this study are commercially available (see catalog numbers above) and show the band of the expected size. In addition, the antibodies directed against the RAD51 paralogs, ZRANB3, BRCA2 and RAD51 were further validated for our approaches using an siRNA directed against the protein of interest and/ or the corresponding knock-out cell line. Validation experiments of the specificity for alpha-tubulin and Ku70 antibodies in western-blotting are displayed on the corresponding manufacturer's websites. The CldU antibody has been validated for immunofluorescence applications, as stated on the Abcam

website. The IdU (BrdU) antibody has been thoroughly validated and used since 1982, as proven by the long list of references provided on the manufacturer's website.

https://www.sigmaaldrich.com/catalog/product/sigma/t9026?lang=de&region=CH&gclid=Cj0KCQjwn7j2BRDrARIsAHJkxmwhLjBkXGE_IUMFtTpAJtSnm3LU817XIeb29HObyX7uuawZhqaQ0CAaAqnYEALw_wcB
https://www.abcam.com/ku70-antibody-2f7f5-ab202022.html#description_images_2
https://www.abcam.com/brdu-antibody-bu175-icr1-proliferation-marker-ab6326.html
https://www.bdbiosciences.com/us/applications/research/apoptosis/purified-antibodies/purified-mouse-anti-brdu-b44/p/347580

Eukaryotic cell lines

Policy information about cell lines

Cell line source(s)	Human U2OS (ATCC); hTERT-RPE1 (ATCC);<br>ZRANB3 proficient and Knock-Out U2OS cells were kindly provided by Dr. David Cortez;<br>RAD51 paralog CRISPSR-Cas9-based Knock-Out U2OS cells have been generated and genetically characterized as recently reported (Reference 44).
Authentication	None of the cell lines were authenticated in house for this manuscript.
Mycoplasma contamination	ZRANB3 cells and human U2OS cells have been tested negative for mycoplasma. hTERT-RPE1 and RAD51 cells have not been tested in our institute.
Commonly misidentified lines<br>(See ICLAC register)	No commonly misidentified lines were used in this study.
